# The prevalence of pediatric asthma hospitalizations at different stages of the COVID-19 pandemic: A systematic review and meta-analysis study protocol

**DOI:** 10.1371/journal.pone.0289538

**Published:** 2023-08-04

**Authors:** Reem Abdelrahim, Zhiwei Gao, Mary Jane Smith, Leigh Anne Newhook

**Affiliations:** 1 Division of Community Health and Humanities, Faculty of Medicine, Memorial University of Newfoundland, St. John’s, Newfoundland and Labrador, Canada; 2 Department of Pediatrics, Memorial University of Newfoundland, St. John’s, Newfoundland and Labrador, Canada; University of Buea, CAMEROON

## Abstract

**Background:**

Asthma is a highly prevalent chronic inflammatory lung disease and is a frequent cause of hospitalization in children. The COVID-19 pandemic has introduced several challenges that have impacted the delivery of care for vulnerable patients, including asthmatic children. Asthmatic children without immediate access to healthcare services can face severe and fatal consequences. Furthermore, various governmental restrictions and viral mutants have been introduced throughout the pandemic, affecting COVID-19 cases and hospitalization rates.

**Objectives:**

To investigate the impact of the COVID-19 pandemic on the prevalence of asthma hospitalizations during various stages of the pandemic. We also aim to compare asthma hospital admissions during the pandemic to pre-pandemic periods.

**Methods and analysis:**

The databases PubMed (MEDLINE), EMBASE, CINAHL, and the Cochrane library will be used to identify relevant articles between the start of the pandemic and the date of the search strategy. Studies will be included if they examine hospital admissions for pediatric (0 to 18 years) asthma patients, regardless of asthma severity, sex, ethnicity or race. Observational retrospective cohort, prospective cohort, and cross-sectional studies will be included. A meta-analysis will be conducted if there are ≥2 articles. Else, a narrative review will be used to report our results.

**Trial registration:**

**PROSPERO registration number:** CRD42022337606.

## Introduction

Asthma is among the leading causes of pediatric hospitalizations and ranks as the most common chronic respiratory illness evident in children [1–3]. According to the World Health Organization (WHO), approximately 300 million people live with asthma worldwide, and that number is expected to increase to 400 million by 2025 [3]. While asthma mortality rates have steadily decreased over the past three decades, asthma morbidity in the form of exacerbations (attacks) or flare-ups continues to affect a large proportion of patients [4]. These attacks are typically induced due to airway constriction and inflammation, causing difficulty in breathing [5, 6]. Although mild asthma exacerbations can be managed at home through inhaled corticosteroids [7], more severe exacerbations may require emergency care [3]. As such, having hospital services readily accessible to asthmatic patients is crucial during these events.

The introduction of the COVID-19 pandemic has introduced new challenges for asthmatic patients [8, 9]. Spearheaded by the Severe Acute Respiratory Syndrome Coronavirus 2 (SARS-CoV-2) virus, the COVID-19 pandemic forced a change in how our healthcare systems operate and, subsequently, the means by which asthmatic patients seek care [10]. During the early stages of the pandemic, it was believed that children were less susceptible to catching serious COVID-19 infections [11]. However, more recent studies have shown that children, particularly infants, can develop severe infections that progress to inpatient and ICU admission [12]. Moreover, the Centre for Disease Control and Prevention (CDC) reports that children with chronic respiratory illnesses, such as asthma, are more vulnerable to developing serious COVID-19 infections that result in hospitalization [13].

Asthmatic exacerbations are frequently triggered by respiratory viruses such as the rhinovirus and the influenza virus [4]. Several studies have shown substantial decreases in the rates of common respiratory viral infections in hospitalized patients during the pandemic [14, 15], which may be attributed to the various social distancing initiatives and government mandates that were implemented as a means to reduce SARS-CoV-2 viral transmission [14]. Furthermore, various studies have reported a significant decrease in general pediatric hospital visits during the pandemic, including those for asthmatic admission [16, 17].

In addition to the government-level restrictions, we have witnessed the periodic emergence of novel viral variants, which have introduced varying degrees of transmissibility, vaccine resistance, and disease severity [18]. The five main variants established as “Variants of Concern” (VOC) by the WHO were the Alpha (B.1.1.7), Beta (B.1.351), Gamma (P.1), Delta (B.1.617.2), and Omicron (B.1.1.529) variants [19]. The two most recent variants, Delta and Omicron, have been the most contagious [19, 20] and have significantly increased the number of COVID-19 hospitalizations [21]. According to researchers at the CDC, the period in which the Delta variant was introduced corresponded to an increase in admissions for children ages 0 to 17 in the United States [22, 23].

COVID-19 mandates, governmental alert levels, and the emergence of viral variants have varied throughout the pandemic timeline. As such, our current systematic review and meta-analysis focuses on investigating how pediatric asthma hospitalizations have changed at different stages of the pandemic. Subsequently, we aim to investigate the risk of admission during the pandemic when compared to the pre-pandemic period for this population.

Specifically, our primary and secondary objectives are as follows:

1. Among pediatric asthma patients (aged 0 to 18), what is the pooled prevalence of non-asthma hospitalizations and hospitalizations due to asthma at 6-month intervals during the pandemic, respectively?
2. Among pediatric asthma patients (aged 0 to 18 years), what is the risk of asthma-related and non-asthma-related hospitalizations during the pandemic when compared to the pre-pandemic period?

## Methods

This systematic review protocol is reported following the Preferred Reporting Items for Systematic Review and Meta-Analyses Protocols (PRISMA-P) checklist [24]. The protocol has been published in PROSPERO International Prospective Register of Systematic Reviews with the registration number CRD42022337606. Any amendments to the protocol will be reflected in our PROSPERO registration.

### Inclusion criteria

#### Participants

Eligible patients are defined as pediatric patients, aged 0 to 18 years, with physician-diagnosed asthma. Following discussion with content experts, we will include studies examining asthma hospital admissions due to upper respiratory tract infections as they are frequent triggers of asthmatic exacerbation and subsequent hospitalization. We will not exclude on the basis of sex/gender, race, or asthma severity. Furthermore, only population-based studies will be included.

#### Exposure

We define the exposure as the indirect effects of the COVID-19 pandemic on asthma hospitalization. The pandemic’s start date will be determined based on the official declaration of the COVID-19 disease outbreak as a pandemic by the WHO. As such, the beginning of the exposure period is defined as March 11th, 2020 [25]. As the pandemic is still ongoing, the entire exposure period is defined as March 11th, 2020, until the date of the searches.

#### Comparator

To address our secondary objective, the comparator period is defined as the pre-pandemic period. Studies which make a comparison at any point in time during the pandemic to the corresponding period of time in the previous year(s) are eligible. Single-year comparator periods (e.g. 2019 only) and multi-year comparator periods (e.g. 2016–2019) are eligible for analysis.

#### Outcome

The outcome of interest is defined as hospitalizations among pediatric asthma patients, regardless of admission reason. All types of hospitalizations (e.g. ICU admission, medicine ward, etc.) and all admission lengths (<24hr and ≥24hr) will be included.

#### Study

Observational studies, such as prospective and retrospective cohorts and cross-sectional studies, will be included. Letters to editors will be included if they contain sufficient information regarding the prevalence of hospital admissions during the pandemic only or between the pandemic and pre-pandemic periods. We will include studies of all language types. Non-English studies will be translated into English using Google Translate where applicable.

### Exclusion criteria

Studies comprising populations aged 19+ only will be excluded. Additionally, studies composed of a mix of pediatric and adult patients will be excluded if the hospital admissions are not differentiated between the two populations. Articles using immediate pre-pandemic timelines (e.g. December 2019 to February 2020) will be excluded since these are periods of time in which the COVID-19 disease outbreak was initiated but not yet declared a pandemic. We will also exclude studies that examine only pre-pandemic pediatric asthma hospital admissions.

Finally, survey studies will be excluded as they are primarily based on self-reported hospital admissions, which may increase the risk of bias. Commentaries, case reports, case series, and modelling studies making predictions on healthcare use will also be excluded at the screening stages.

### Information sources

A comprehensive search strategy (S1 Appendix) was developed in collaboration with a health sciences librarian and following the Peer Review Electronic Search Strategy (PRESS) guidelines [26]. The databases PubMed (MEDLINE), EMBASE, CINAHL, and the Cochrane Library were searched to identify relevant articles. Medical Subject Headings (MeSH) terms and keyword search terms related to the previously specified PECO items were used to enhance the search strategy. To implement a sensitive search, no restrictions on the language or publication type were applied. Articles were searched from the start of the pandemic (2020) until the date of the search strategy. Additionally, a hand search of potential articles referenced in the identified primary articles will be conducted to identify relevant articles not revealed using the search strategy. Authors of conference abstracts will be contacted to identify additional studies/data. If we cannot attain a full-text version of the abstracts, the study will be excluded.

### Study selection and search strategy

**Fig 1:**
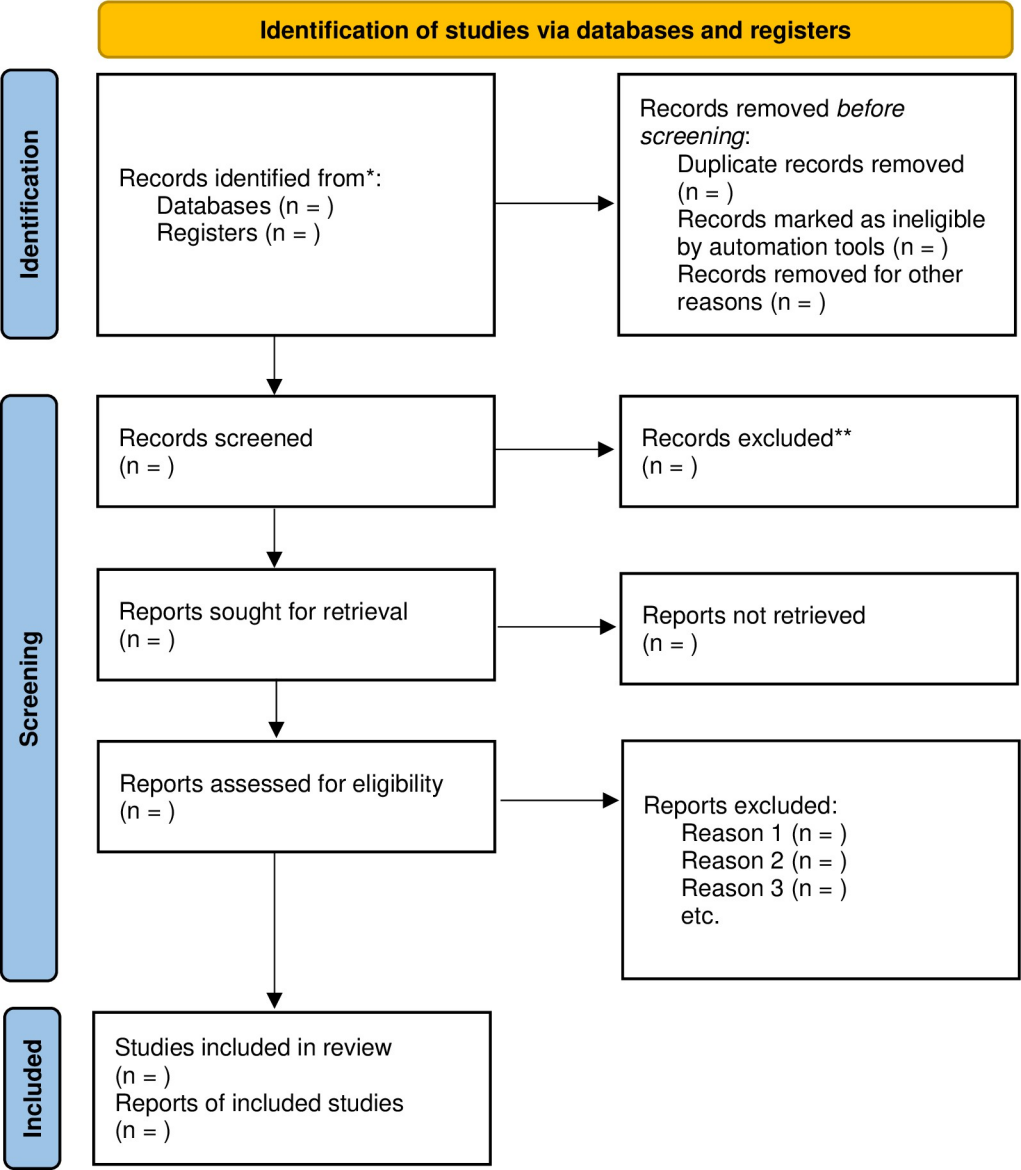
PRISMA flow diagram template from Page et al. [28].

The search strategy results from all the databases and hand searching will be imported into the Covidence software [27] for screening, and duplicates will be removed within the software. Title and abstracts will be screened by at least two reviewers following the inclusion and exclusion criteria discussed in the earlier sections. Any disagreements between the reviewers will be discussed until a consensus is reached. If necessary, a third reviewer (ZG or LN) will be consulted for a final decision. Full-text screening will be completed by the same two independent reviewers, and when necessary, a third author (ZG or LN) will be consulted to reach a final decision. At this stage, any excluded studies will be accompanied by recorded reasons for exclusion. Further, if there are multiple articles reporting results from the same study, we will only use the most recent article in our review. The entire two-stage study screening and selection process will be displayed using the PRISMA flowchart (Fig 1).

### Data extraction

Two independent viewers with research experience related to pediatric asthma will conduct the data extraction process using a pre-designed data extraction sheet based on the Joanna Briggs Institute (JBI) Manual for Systematic Reviews and Meta-Analyses [29] and the Strengthening the Reporting of Observational Studies in Epidemiology (STROBE) [30]. Disagreements will be resolved through discussion or by consulting a third author to reach a final decision. We will extract the following data:

1. Study characteristics: (e.g. Study authors, publication year, study design, location, single vs. multi-center)
2. Participant characteristics: (e.g. participant age range, sex, asthma diagnosis description)
3. Outcomes measured
4. Exposure data: setting information (e.g. exposure description, timeline studied), sources of data, and the data analysis methods used

Study authors will be contacted for missing information. Authors will be contacted a maximum of three times for data. If no response is received following contact, the study will be excluded.

### Risk of bias

The Risk of Bias assessment will be conducted using the Risk of Bias In Non-randomised Studies—of Exposure (ROBINS-E) tool [31], a modified version of the ROBINS-I (intervention) tool [32]. This tool will be applied for cohort and case-control studies. We will follow the guidance provided by Morgan and colleagues [31] in using this tool, in addition to the guidance provided for the ROBINS-I tool [32], given there is much overlap between the domains measured. For the purposes of the current systematic review and meta-analysis, the tool will be examined in reference to exposures instead of interventions. Each decision will be supplemented with a quote from the study to justify the decision. Domains will be scored as either low, moderate, serious, or critical risk of bias, or no information. The overall risk of bias will be determined following the guidance provided in the ROBINS-I guidance tool [32] and the ROBINS-E guidance publication by Morgan et al. [31].

The JBI critical appraisal tool for cross-sectional studies [33] will be used to analyze cross-sectional studies. Two reviewers will independently assess the risk of bias for all the included studies. If a conflict arises, the reviewers will discuss until a consensus is reached. Otherwise, a third author will be consulted to reach a final decision.

### Synthesis of results

#### Data synthesis

A meta-analysis will be conducted if two or more studies are included by the end of the study screening stages. The Review Manager Software (RevMan version 5.4.1, Cochrane Collaboration, Oxford, UK) [34] will be used to perform the quantitative data analysis. A fixed-effects model (Mantel-Haenszel) will be used if the statistical heterogeneity is not significant. Otherwise, the random-effects model (DerSimonian and Laird) will be used. Effect sizes will be presented as a prevalence estimate to address our primary objective and as an odds ratio with 95% confidence intervals to address our secondary objective. We will use the I^2^ statistic and the Q-test (significance level of 0.10) to inspect the level of heterogeneity. The level of heterogeneity is defined as described by Guyatt and colleagues, whereby an I^2^ of 0–40% is considered low, 30–60% as moderate, 50–90% as substantial, and 75–100% as considerable heterogeneity [35]. If there is substantial heterogeneity (I^2^ ≥50%), the source of heterogeneity will be further explored through subgroup analysis of the clinical (e.g. population characteristics, exposure period) and methodological (e.g. study design) differences between the studies. We will also examine the direction and magnitude of the individual-study effect sizes to assess if calculating a pooled estimate is suitable.

If the quantitative analysis is not feasible due to substantial heterogeneity (I^2^ is ≥50%), the data will be summarized and explained using a narrative synthesis and tabular synthesis of the study findings and characteristics. The Synthesis Without Meta-Analysis (SWiM) guidelines [36] will be used as a reporting guideline to present the findings qualitatively.

#### Subgroup analysis

We will conduct a subgroup analysis between geographic regions (Africa, Asia, Europe, North America, and South America) since the magnitude and impact of the pandemic show geographical variations [37]. Additionally, a subgroup analysis based on asthma severity and control may be conducted.

### Quality of evidence

The quality of evidence from the meta-analysis will be determined using the GRADE approach developed by Guyatt and colleagues [35]. Although the articles included in the meta-analysis will be non-randomized studies and observational, we will set the quality of evidence as “high” by default since the risk of bias will be analyzed using the ROBINS-E tool, which accounts for confounding and selection bias [31, 35, 38]. Quality will be rated down for the first five GRADE domains and rated up for the last three domains. The risk of bias domain will be downgraded based on the combined ROBINS-E judgment of all the studies (Low, Moderate, Serious, or Critical) for both the confounding and selection bias domains.

We will upgrade the certainty of evidence by one point if there is a large magnitude of effect, a significant dose-response gradient, and plausibility that residual confounders cannot explain the outcome effects. The GRADE certainty of evidence will be rated as high, moderate, low, or very low. Given the observational nature of the studies, and potential residual confounding, we do not anticipate attaining a high or moderate level of certainty [39].

## Supporting information

S1 Appendix Search strategy. (DOCX)

S2 Appendix PRISMA-P checklist. (DOCX)

S1 File Minimal dataset. (DOCX)
