# Supplementary material for: The prevalence of pediatric asthma hospitalizations at different stages of the COVID-19 pandemic: A systematic review and meta-analysis study protocol
Source: PLoS One. 2023 Aug 4;18(8):e0289538. doi: 10.1371/journal.pone.0289538 (PMC10403069; doi:10.1371/journal.pone.0289538)
Supplement: S1 Appendix — (DOCX) [file pone.0289538.s001.docx]

| PubMed May 3, 2022 | | |
| --- | --- | --- |
| 9 | #4 and #5 and #6 and #7  Filters: Human, from 2020-2022 | 624 |
| 8 | #4 and #5 and #6 and #7 | 915 |
| 7 | "Hospitalization"[Mesh] OR hospitalization OR hospitalized OR hospitalisation OR hospitalised OR admission OR admit* Sort by: Publication Date | 6 255 634 |
| 6 | Search: pediatric* OR paediatric* OR child* OR adolescen* OR infant* OR teen* Sort by: Publication Date | 4 910 137 |
| 5 | Search: "Asthma"[Mesh] OR asthma* Sort by: Publication Date | 210 686 |
| 4 | #1 OR #2 OR #3 | 555 304 |
| 3 | Search: coronavirus OR covid-19 OR "sars-cov-2" OR "SARS virus" OR covid OR "severe acute respiratory virus 2" OR delta OR omicron Sort by: Publication Date | 452 792 |
| 2 | Search: (("Disease Outbreaks"[Mesh]) OR "Epidemics"[Mesh]) OR "Pandemics"[Mesh] Sort by: Publication Date | 183 055 |
| 1 | Search: "COVID-19"[Mesh] OR "SARS-CoV-2"[Mesh] OR "SARS-CoV-2 variants" [Supplementary Concept] OR "SARS Virus"[Mesh] OR "Coronavirus"[Mesh] OR "Coronavirus Infections"[Mesh] Sort by: Publication Date | 174 866 |

| CINAHL Plus (EBSCO) May 3, 2022 | | |
| --- | --- | --- |
| 10 | #4 and #5 and #6 and #9  Filters: Human, from 2020-2022 | 53 |
| 9 | #7 OR #8 |  |
| 8 | hospitalization or hospitalized or hospitalisation or hospitalised or admission or admit* | 254 511 |
| 7 | (MH "Hospitalization+") OR (MH "Hospitals, Pediatric") | 6 255 634 |
| 6 | pediatric* OR paediatric* OR child* OR adolescen* OR infant* OR teen* | 1 376 561 |
| 5 | (MH "Asthma") OR asthma* | 49 384 |
| 4 | #1 OR #2 OR #3 | 132 987 |
| 3 | coronavirus OR covid-19 OR "sars-cov-2" OR "SARS virus" OR covid OR "severe acute respiratory virus 2" OR delta OR omicron | 105 238 |
| 2 | MH “Disease Outbreaks: | 41 266 |
| 1 | (MH "COVID-19") OR (MH "COVID-19 Pandemic") OR (MH "SARS-CoV-2") OR (MH "Coronavirus") OR (MH "Coronavirus Infections") OR (MH "SARS Virus") | 57 954 |

| Cochrane (Wiley) June 3, 2022 | | |
| --- | --- | --- |
| 8 | #3 AND #4 AND #5 AND #7 | 91 |
| 7 | (covid-19 OR sars-cov-2 OR “sars-cov-2 variants” OR coronavirus OR “coronavirus infections” OR epidemic* OR pandemic* OR “disease outbreak” OR “disease outbreaks” OR covid OR “severe acute respiratory virus” OR delta OR omicron) | 23240 |
| 6 | #2 AND #3 AND #4 AND #5 | 25 |
| 5 | hospitalization OR hospitalisation OR hospitalized OR hospitalised OR admission OR admit* | 101970 |
| 4 | pediatric* OR paediatric* OR child* OR infant* OR teen* | 324281 |
| 3 | asthma* | 38394 |
| 2 | (covid-19 OR sars-cov-2 OR “sars-cov-2 variants” OR coronavirus OR “coronavirus infections” OR epidemic* OR pandemic* OR “disease outbreak” OR “disease outbreaks” OR covid OR “severe acute respiratory virus” OR delta OR omicron):ti,ab,kw (Jan 2020 - May 2022) | 13407 |
| 1 | covid-19 | 10640 |

| EMBASE May 3, 2022 | | |
| --- | --- | --- |
| 10 | #4 AND #5 AND #6 AND #9 AND ([article]/lim OR [article in press]/lim OR [conference abstract]/lim OR [conference paper]/lim OR [preprint]/lim) AND [embase]/lim AND [2020-2022]/py | 444 |
| 9 | #7 OR #8 | 1 372 050 |
| 8 | hospitalization OR hospitalized OR hospitalisation OR hospitalised OR admission OR admit* | 1 352 016 |
| 7 | 'hospitalization'/exp OR 'pediatric hospital'/exp | 484 805 |
| 6 | pediatric* OR paediatric* OR child* OR adolescen* OR infant* OR teen* | 5 548 439 |
| 5 | "Asthma"[Mesh] OR asthma* | 362 487 |
| 4 | #1 OR #2 OR #3 | 551 352 |
| 3 | coronavirus OR covid-19 OR "sars-cov-2" OR "SARS virus" OR covid OR "severe acute respiratory virus 2" OR delta OR omicron | 417 638 |
| 2 | 'epidemic'/exp OR 'pandemic'/exp | 238 811 |
| 1 | 'coronavirus disease 2019'/exp OR 'severe acute respiratory syndrome coronavirus 2'/exp OR 'sars-cov-2 variant 501y.v1'/exp OR 'sars coronavirus'/exp OR 'coronavirinae'/exp OR 'coronavirus infection'/exp | 260 381 |
