# Supplementary material for: The prevalence of pediatric asthma hospitalizations at different stages of the COVID-19 pandemic: A systematic review and meta-analysis study protocol
Source: PLoS One. 2023 Aug 4;18(8):e0289538. doi: 10.1371/journal.pone.0289538 (PMC10403069; doi:10.1371/journal.pone.0289538)
Supplement: S1 File — (DOCX) [file pone.0289538.s003.docx]

1. Abe K, Miyawaki A, Nakamura M, Ninomiya H, Kobayashi Y. Trends in hospitalizations for asthma during the COVID-19 outbreak in Japan. J Allergy Clin Immunol Pract. 2021;9(1):494-496.
2. Alshengeti A, Alahmadi H, Barnawi A, Alfuraydi N, Alawfi A, Al-Ahmadi A, et al. Epidemiology, clinical features, and outcomes of coronavirus disease among children in Al-Madinah, Saudi Arabia: A retrospective study. Int J Pediatr Adolesc Med. 2022;9(2):136-142.
3. Alsulaiman JW, Kheirallah KA, Ajlony MJ, Al-Tamimi TM, Khasawneh RA, Al-Natour L. Paediatric asthma exacerbation admissions and stringency of non-pharmaceutical interventions: Results from a developing country. Int J Clin Pract. 2021;75(9):e14423
4. Antoon JW, Grijalva CG, Thurm C, Richardson T, Spaulding AB, Teufel RJ 2nd, et al. Factors Associated With COVID-19 Disease Severity in US Children and Adolescents. J Hosp Med. 2021;16(10):603-610.
5. Bartha I, Bernaola M, Escudero C, Rodríguez Del Río P, Bazire R, Ibáñez Sandín MD. COVID-19 and childhood asthma: Analysis of a pediatric referral hospital. Pediatr Allergy Immunol. 2022;33(3):e13757.
6. Belhadjer Z, Méot M, Bajolle F, Khraiche D, Legendre A, Abakka S, et al. Acute Heart Failure in Multisystem Inflammatory Syndrome in Children in the Context of Global SARS-CoV-2 Pandemic. Circulation. 2020;142(5):429-436.
7. Bun S, Kishimoto K, Shin JH, Maekawa T, Takada D, Morishita T. Impact of the COVID-19 pandemic on asthma exacerbations in children: A multi-center survey using an administrative database in Japan. Allergol Int. 2021;70(4):489-491.
8. Kara AA, Böncüoğlu E, Kıymet E, Arıkan KÖ, Şahinkaya Ş, Düzgöl M, et al. Evaluation of predictors of severe-moderate COVID-19 infections at children: A review of 292 children. J Med Virol. 2021;93(12):6634-6640.
9. Kumar A, Taitt J, Sign P. The impact of COVID-19 pandemic on the burden and the pattern of hospitalization from COVID-19 unrelated illnesses among children in barbados – A preliminary report from an ongoing study. Int Arch Health Sci. 2021; 8(3):212-216
10. Liljestrom T, Bauer SC, Moral F, Preloger E, Chelampath M. Pediatric Respiratory Illness Hospitalizations Prior to COVID-19 and During the First Year of the COVID-19 Pandemic in Southeast Wisconsin. WMJ. 2022;121(1):54-57.
11. Markham JL, Richardson T, DePorre A, Teufel RJ, Hersh AL, Fleegler EW, et al. Inpatient Use and Outcomes at Children's Hospitals During the Early COVID-19 Pandemic. Pediatrics. 2021;147(6):e2020044735.
12. Sano K, Nakamura M, Ninomiya H, Kobayashi Y, Miyawaki A. Large decrease in paediatric hospitalisations during the COVID-19 outbreak in Japan. BMJ Paediatr Open. 2021;5(1):e001013.
13. Sheehan WJ, Patel SJ, Margolis RHF, Fox ER, Shelef DQ, Kachroo N,et al. Pediatric asthma exacerbations during the COVID-19 pandemic: Absence of the typical fall seasonal spike in Washington, DC. J Allergy Clin Immunol Pract. 2021;9(5):2073-2076.
14. Cabrera SL, Zamora TA, Guerreiro NMC, et al. Clinical-epidemiological characterization of 77 children and adolescents infected with SARS-CoV-2 coronavirus. Rev Cubana Pediatr. 2021;93(1):1-14.
15. Tagarro A, Cobos-Carrascosa E, Villaverde S, Sanz-Santaeufemia FJ, Grasa C, Soriano-Arandes A, et al. Clinical spectrum of COVID-19 and risk factors associated with severity in Spanish children. Eur J Pediatr. 2022;181(3):1105-1115.
16. Yildiz E, Cigri E, Dincer Z, Narsat MA, Calisir B. High Neutrophil/Lymphocyte Ratios in Symptomatic Pediatric COVID-19 Patients. J Coll Physicians Surg Pak. 2021;31(7):S93-S98.
17. Zee-Cheng JE, McCluskey CK, Klein MJ, Scanlon MC, Rotta AT, Shein SL, et al. Changes in Pediatric ICU Utilization and Clinical Trends During the Coronavirus Pandemic. Chest. 2021;160(2):529-537.
